# Clinical Lecture on the Kousso

**Published:** 1851-03

**Authors:** 


*Clinical Lecture on the Kousso.*—[Dr. Budd having obtained a supply of this medicine, tested its properties in several cases, upon which he subsequently made the following comments:]

The medicine has been given in the morning, before breakfast, which is the best time for administering all remedies for tape-worm, as the small intestine which the worm inhabits is then more empty than at other times; the worm, consequently, less likely to be sheathed, and protected from the action of the drug, by the other contents of the bowel.

The powder has been infused for ten minutes in three-fourths of a pint of hot water. The infusion has then been stirred, and the whole drunk.

One of the patients, under the care of Dr. Todd, a woman advanced in pregnancy, vomited about half of the dose; but what remained in the stomach destroyed the worm. Two of the other patients had a slight feeling of nausea for ten minutes or a quarter of an hour, after taking the medicine. Another was several times purged by it; another, the woman to whom we first gave it, had headache after taking it, and ascribed to it a diuretic effect. The rest felt no uneasy sensation whatever from the medicine.

In the cases that fell under my own care, I ordered the patients to live sparingly, and to take a seidlitz powder, or a dose of castor-oil, the day before taking the kousso, for the sake of emptying the bowels, and so leaving the worm more exposed. This is a precaution which it is well to take before administering any of the remedies for tape-worm. They all act directly on the worm, and to take effect must, of course, be brought in contact with it.

I also ordered the patients to take a seidlitz powder, or a dose of castor-oil, after the kousso, to carry the medicine down to the worm, and to expel the dead or enfeebled worm from the bowels. This expedient, also, is equally applicable to other remedies for the tape-worm, and especially to turpentine, which, if it remains long in the stomach, or passes slowly through the bowels, gets absorbed and irritates the kidneys and bladder, producing strangury and bloody urine, and may not reach the worm in quantity sufficient to kill it.

Tape-worms are very tenacious of life. They are seldom voided entire without the aid of medicines that act especially upon them. Single joints often come away, and pieces two or three feet long are now and then voided, but it very seldom happens, unless after medicine, that a portion comprising the head is thus passed. This portion remains and grows again, so that a person is often plagued with the parasite for years. One of our patients, Sarah W——, had been so plagued for sixteen years, during which she told us that she had seldom gone a week without passing joints of the worm. She had taken turpentine, and the bark of the pomegranate root, and some years ago, when she was at Fort Beaufort, at the Cape of Good Hope, she took the root of a plant used by the natives as a remedy for tape-worm, which she says is called there "Cacay." She describes this root as being round, and, when scraped, white, like a turnip, and sweet to the taste. This medicine had very little effect. The turpentine and the pomegranate brought away long pieces of the worm, but the head and the portion near it remained, and the worm grew again. The worm was expelled after the kousso in different portions, on the 11th and 12th of April, and she has since had none of the symptoms which she attributed to it. She is still (May 31st) in the hospital, for prolapsus of the uterus, which she had long had, but she feels convinced that the worm is entirely destroyed.

Another case, that shows more strikingly still the tenacity of life of the tape-worm, is that of Samuel P——. He first passed joints of the worm seven years ago. In September last, he was brought into the hospital with cholera, and on the day of his admission passed a portion of the worm two yards in length. He remained in the hospital three weeks, on account of the cholera. Some time after this, he again passed joints of the worm, and to get rid of it came to the hospital as an out-patient. Turpentine and castor-oil were given him, and brought away a long piece of the worm. The ailments, which he attributed to the worm, were much relieved for a time. They then became more severe; joints of the worm were again passed, and in January last he came for the second time to the hospital to be ridded of the worm. Turpentine and castor-oil were given him, as before; a long piece of the worm was again expelled, but the creature was not destroyed; so that here the worm had existed seven years, had kept its place during the terrible commotion and flooding of the intestines in cholera, and had escaped destruction by two doses of turpentine. P—— took the kousso on the 3d of May, and the next morning voided the worm, which was ten yards long.

In all cases in which the kousso has been given in the hospital, the head of the worm, or the taper portion near the head, has been found, so that there is reason to suppose that the creature has been entirely destroyed. The action of the medicine is very speedy. In one of the cases, in which the bowels were slow to move, the worm was expelled, in different portions, in the two days succeeding that in which the kousso was taken; in all the other cases it was expelled the same day, and in several of them after the lapse of only three or four hours.

The result of our experience, then, is, that the kousso is a very effectual remedy for tape-worm; and that in its action it is both speedy and safe.

I have had little experience of the male fern. But the kousso is certainly much more efficacious than the pomegranate; and much more efficacious, as well as less disagreeable, and safer than turpentine. There is a considerable demand for it at the hospital, which, on account of the cost, we cannot satisfy. I trust, however, now that its efficacy is known in this country, which has commercial relations with every part of the world, that a fresh supply will soon be brought here, and that we shall have it at a reasonable rate.

From one of our patients two worms, one very large, and the other much smaller, were expelled by the kousso. It sometimes happens that as many as three or four of these worms are found together; but such an event is very uncommon. The tape-worm requires no mate, and generally leads a solitary life; and, hence, has been called by the French, *ver solitaire*—the solitary worm.

In the cases which fell under my own care at the hospital, notes were taken of the ailments which the patients attributed to the worm. Most of these persons had some unpleasant sensations referred to the stomach and bowels, such as a feeling of sickness or nausea, especially on first getting up in the morning; pain, or a sense of sinking at the epigastrium; flatulence; a feeling at times as if a worm were moving in the bowel. It is worthy of remark, that in none of them was there diarrhœa. The appetite is very variable—in some, much impaired, in others, craving.

Two or three of the patients had a troublesome cough; and one, annoying palpitation; which were ascribed to the worm. Some of them complained, also, of unpleasant sensations in the head; giddiness or drowsiness felt during the day, and especially after meals; and almost all complained, more or less, of weakness, and lassitude, and incapacity of work.

The most constant of the symptons were a feeling of lassitude, a sense of nausea on first getting up in the morning, and drowsiness felt during the day. But symptoms such as these are of very little use in diagnosis. Persons who have tape-worm generally discover the fact by the passing of joints. The worm seems to grow rapidly; and the tail-joints, as they attain their full development, drop off, and are voided with the other contents of the bowel. Probably no person suffers very long from tape-worm, without voiding some of these joints. The existence of the worm cannot be safely inferred, and the remedies for it cannot be given with a fair chance of success, unless joints have been passed. It is a common belief that while the joints are dropping, the worm is feeble, and that the medicines which kill it act more surely soon after joints have been passed.

[Numerous cases have been recorded by Mr. Armstrong, ('Lancet') and others, which fully confirm the superiority of this medicine over those usually given in tape-worm.] —*Ib.*

